# First report of concomitant cryptococcal meningitis and anti-NMDAR encephalitis

**DOI:** 10.1016/j.bbih.2020.100036

**Published:** 2020-01-09

**Authors:** Sofia R. Valdoleiros, Margarida Calejo, António Marinho, Ana Martins da Silva, Olga Vasconcelos, Maria João Gonçalves, Rui Sarmento e Castro

**Affiliations:** a Department of Infectious Diseases, Centro Hospitalar Universitário do Porto, Largo Prof. Abel Salazar, 4099-001, Porto, Portugal; b Department of Neurology, Centro Hospitalar Universitário do Porto, Largo Prof. Abel Salazar, 4099-001, Porto, Portugal; c Department of Internal Medicine, Centro Hospitalar Universitário do Porto, Largo Prof. Abel Salazar, 4099-001, Porto, Portugal; d Unit for Multidisciplinary Research in Biomedicine, Instituto de Ciências Biomédicas Abel Salazar, University of Porto, 4099-001, Porto, Portugal

**Keywords:** Anti-N-Methyl-D-Aspartate receptor, Anti-N-Methyl-D-Aspartate receptor encephalitis, Cryptococcosis, Cryptococcal meningitis, Cryptococcus neoformans, Sarcoidosis

## Abstract

Anti-N-methyl-D-aspartate receptor (NMDAR) encephalitis is an autoimmune disorder, seen most often in young adults and children, triggered by tumors or infections. We report a case of cryptococcal meningitis in a patient with sarcoidosis, presenting prominent neuropsychiatric symptoms, electroencephalographic features of autoimmune encephalitis and positive anti-NMDAR antibodies in the cerebrospinal fluid, raising the hypothesis of an infectious immune-mediated mechanism triggering the production of anti-NMDAR antibodies. Since anti-NMDAR encephalitis is potentially fatal and has significant morbidity, further descriptions of its etiological associations are essential to early identification and prompt treatment.

## Highlights

• Cryptococcal meningitis is a rare complication of sarcoidosis and CD4 lymphopenia.
• Anti-NMDAR encephalitis is an autoimmune disorder triggered by tumors or infection.
• A case of concomitant cryptococcosis and anti-NMDAR encephalitis is presented.

## 1 Introduction

Sarcoidosis is a multisystem granulomatous disease of unknown etiology. Lymphopenia is common (Crouser et al., 2010; Jamilloux et al., 2015; Morell et al., 2002; Yanardag et al., 2002) and T-cell mediated immunity has been shown to be impaired (Adams and Gibson, 2016; Bernard et al., 2013; Dhote et al., 2009; Jamilloux et al., 2015; Leonhard et al., 2016; Miyara, 2006; Ross and Katz, 2002). Albeit infrequent, cryptococcosis is a known complication (Bernard et al., 2013; Jamilloux et al., 2015; Leonhard et al., 2016; Peret et al., 2014), as T-cell immunity is the predominant pathway for protection against infection by *Cryptococcus neoformans* (Bernard et al., 2013; Riha and Allen, 2004). The frequent administration of immunosuppressive therapy to patients with sarcoidosis, particularly steroids, may potentiate the already present relative susceptibility to cryptococcosis (Baughman and Lower, 2005; Dhote et al., 2009; Mehrany et al., 2002; Peret et al., 2014). Nevertheless, cryptococcal meningitis can develop even in the absence of immunosuppressive therapy (Adams and Gibson, 2016; Girard et al., 2004; Jamilloux et al., 2015; Leonhard et al., 2016; Peret et al., 2014).

Anti-N-methyl-D-aspartate receptor (NMDAR) encephalitis, an autoimmune disorder, is not uncommon and its frequency has come to rival that of viral encephalitis (Gable et al., 2012). First described in 2007, there is still insufficient understanding of its etiologies and pathogenesis. Whilst mostly associated to neoplasms (Gable et al., 2009; Liu et al., 2017; Lynch et al., 2018; Venkatesan and Adatia, 2017), some infections appear to precede it in a large set of individuals, which pathogenesis is now an active area of investigation (Venkatesan and Benavides, 2015). Mechanisms by which infections may lead to CNS autoimmunity are manifold (Venkatesan and Benavides, 2015). There is significant evidence that herpes simplex can trigger anti-NMDAR encephalitis (Armangue et al., 2013, 2014; Prüss et al., 2012; Venkatesan and Benavides, 2015); there are also reports of possible links to Varicella-Zoster virus (Dalmau et al., 2011; Schabitz et al., 2014), influenza virus, Japanese Encephalitis virus (Ma et al., 2017) and Human Immunodeficiency Virus (HIV) (Arboleya et al., 2016; Patarata et al., 2016), but, to our knowledge, it has not yet been reported any association to fungal infection. Although potentially treatable, anti-NMDAR encephalitis can lead to death if untreated and is associated with significant morbidity (Lynch et al., 2018; Venkatesan and Benavides, 2015). Hence, further understanding of its etiologies is essential to allow early identification and timely treatment.

## 2 Case report

A previously healthy 39-year-old man received a diagnosis of pulmonary sarcoidosis on September 2016. He was started on prednisolone (40 ​mg per day) on December 2016, but soon tapering was commenced; on July 2017, he was on 10–15 ​mg per day on alternate days.

Progressively worsening headaches, accompanied by photophobia and changes in sleep (alternation between insomnia and somnolence/prostration) developed in September 2017 and motivated an increase in prednisone to 40 ​mg per day for a suspected diagnosis of neurosarcoidosis. At this time, CT scan and MRI of the brain were unremarkable. Four weeks later, changes in behavior and speech, confusion, memory difficulties and visual hallucinations, with periods of psychomotor agitation, without fever, appeared. The patient was medicated with amitriptyline, escitalopram, olanzapine and clonazepam, without improvement. On October 2017, an episode of loss of consciousness, with urinary incontinence and unresponsiveness, without apparent tonic-clonic movements, was described by the patient’s wife. A few days later he was admitted to our hospital. Neurological examination at the Emergency Department (day 0) revealed inattention and disorientation, with psychomotor retardation and no verbal or motor initiative; speech was non-fluent, with hesitations in naming and repeating; myoclonus of the upper limbs, postural tremor and gait ataxia were observed. Meningeal signs were absent.

On blood panel, C-reactive protein was within normal range (4.68 ​mg/L) and white blood cells were elevated (15.49 ​× ​10^9^/L), with neutrophilia (14.30 ​× ​10^9^/L) and lymphopenia (0.36 ​× ​10^9^/L). Immunophenotyping of lymphocytes disclosed an absolute CD4^+^, CD8^+^ and CD3^+^ T cells lymphopenia, with 0.066 ​× ​10^9^/L, 0.134 ​× ​10^9^/L and 0.180 ​× ​10^9^/L, respectively. CD19 ​^+^ ​B cells lymphopenia was also present (0.053 ​× ​10^9^/L). Natural killer cells were within normal range (0.375 ​× ​10^9^/L). Relative CD4 and CD8 T cells lymphopenia (with no available absolute count) of 5.6% and 21.1%, respectively, were already present in 2016 (before the diagnosis of sarcoidosis and without steroids). HIV serology was negative, immunoglobulins were normal and there was no complement consumption.

Nucleocapsular focal hypodensities, without mass effect or hydrocephalus, were seen on the admission CT scan. A lumbar puncture was performed, revealing a cerebrospinal fluid (CSF) opening pressure above 40 cmH_2_O. CSF analysis disclosed pleocytosis, with polymorphonuclear predominance, glucose consumption and elevated proteinorrachia (Table 1). Angiotensin-converting enzyme (ACE) dosing in CSF was inferior to the detection limit. Amplification of nucleic acids from CSF was negative for *Mycobacterium tuberculosis*, Cytomegalovirus (CMV), John Cunningham virus (JCV), Epstein-Barr virus (EBV), Herpes-6, Herpes simplex 1 and 2. CSF venereal disease research laboratory (VDRL) was also negative. Detection of *Cryptococcus neoformans* in the CSF prompted combined therapy with liposomal formulation of amphotericin B and flucytosine. The patient was admitted to the Infectious Diseases’ ward.

**Table 1:** Cerebrospinal fluid cytochemical data of the patient.^a^

Hospital Day after Admission	Glucose (mmol/L)	Proteins (mg/L)	White Cells (x 10^6^/L)	Neutrophils (x 10^6^/L)	Lymphocytes (x 10^6^/L)	Monocytes (x 10^6^/L)
Day 0	0.67	1860	330	257	53	20
Day 2	0.94	1290	266	194	37	24
Day 8	2.72	920	140	48	65	24
Day 16	2.94	1050	42	6	36 mononuclear cells^b^	
Day 27	2.50	750	10	4	6 mononuclear cells^b^	
Follow-up (6 months after admission)	3.00	770	25	13	12 mononuclear cells^b^	

a Reference ranges: glucose, 2.60–4.51 ​mmol/L; proteins, 0–400 ​mg/L; white cell count, 0–5 ​cells x 10^6^/L.

b Distribution of lymphocytes, monocytes and eosinophils not available.

**Fig. 1:**
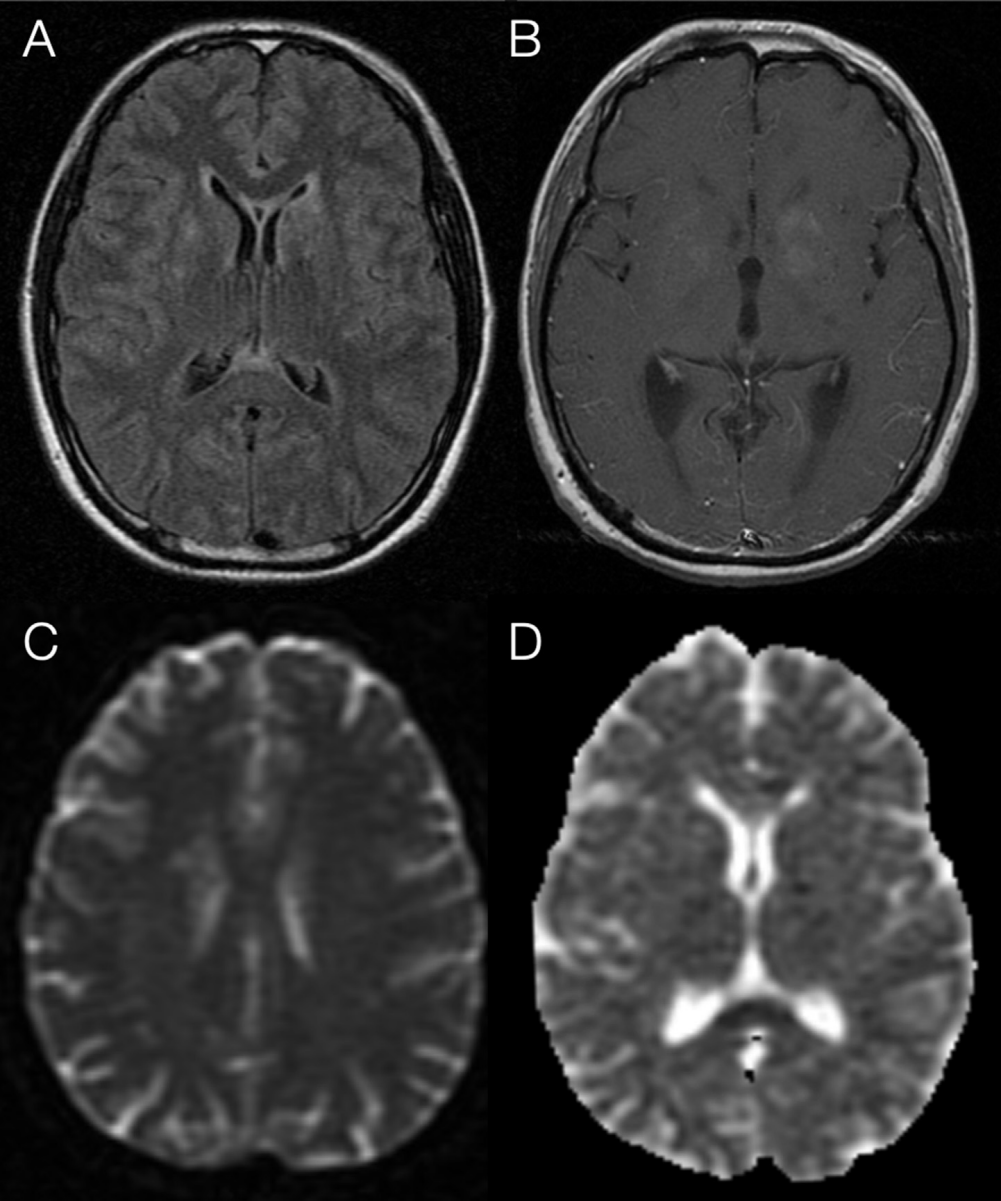
**A, B:** Brain MR imaging showing basal ganglia T2 hyperintense **(A)** and T1 contrast-enhancing areas **(B)**, suggestive of cryptococcal lesions. **C, D:** Subcortical white matter diffusion weighted imaging (DWI) hyperintensities, possibly related to small vessel vasculitic phenomena. **D:** Apparent diffusion coefficient (ADC) map showing hypointensity in the splenium of corpus callosum, as previously described in cases of cryptococcal encephalitis.

**Fig. 2:**
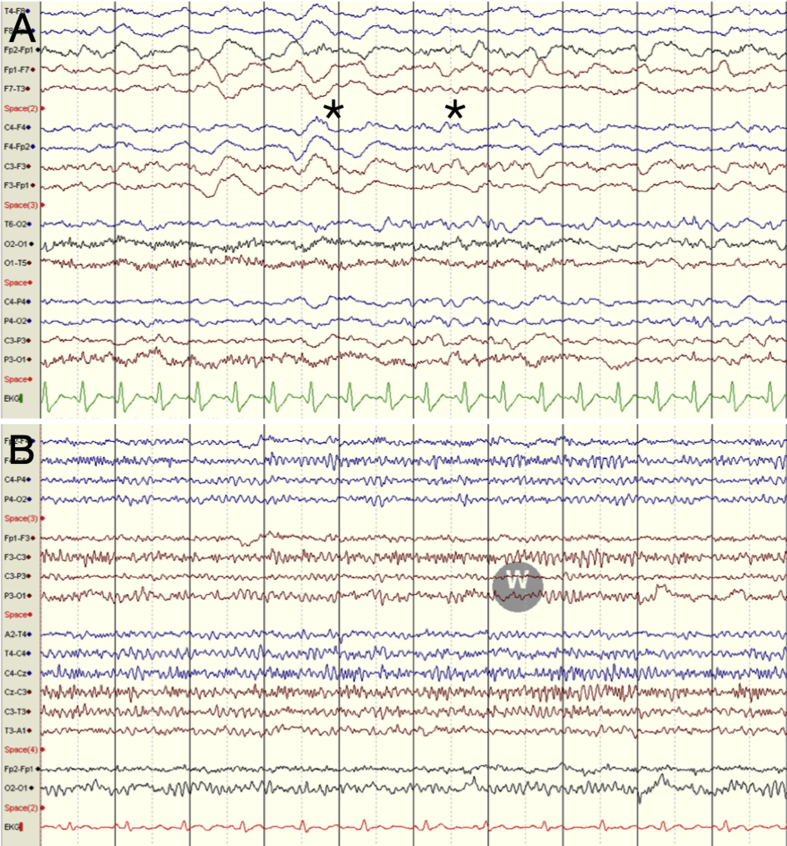
**SupplementaryData. A:** EEG showing global slowing with occasional inscription of delta waves with superimposed beta activity in a ‘delta brush pattern’ (asterisks*). Occasional posterior paroxystic activity. **B:** Post-treatment EEG, with normalized, well-structured alpha activity.

On day 1, given the patient’s immunodepression and since there was no evidence of neurosarcoidosis, prednisolone was reduced to 10 ​mg per day to enhance response against infection. However, despite cytochemical improvement of CSF (Table 1) and normalization of CSF pressure with daily performance of evacuating lumbar punctures, a progressive depression of consciousness was observed since day 7. Brain magnetic resonance imaging (MRI) revealed T1 contrast-enhancing focal areas in the basal ganglia compatible with cryptococcal lesions and small acute ischemic lesions in the subcortical white matter, in possible relation with a vasculitic process (Fig. 1). The patient remained afebrile, but C-reactive protein increased (149 ​mg/L), without leukocytosis. On day 10, an electroencephalogram (EEG) showed global slowing, with ‘delta brush’ activity and paroxysmal posterior focal activity (Fig. 2A, Supplementary Data). The patient was started on levetiracetam (500 ​mg twice daily), but his neurological condition showed no improvement. Day 13’s revaluation EEG exhibited slow and monotonous activity, with registration of slow delta bilateral sequences and no paroxysmal activity, congruent with a severe encephalopathic process of non-specific etiology. He also developed sustained hypotension with sinus tachycardia (systolic arterial pressure, 77–90 ​mmHg; diastolic arterial pressure, 39–55 ​mmHg; heart rate, 115–138 beats per minute) that did not respond to fluids or hydrocortisone for two days, and required admission to Intermediate Care Unit. IgG anti-NMDAR antibodies, directed to the NR1 subunit, were positive in the CSF (negative in serum). Elevated intrathecal IgG synthesis was registered (1.92 ​μmol/L to an upper limit of normal of 0.27 ​μmol/L), and eight CSF-restricted oligoclonal bands were found. Thoraco-abdomino-pelvic CT scan, a testicular ultrasound and a whole-body PET scan were performed, with no evidence of neoplasia.

Treatment for anti-NMDAR encephalitis was instituted. Since it was feared that intensive immunosuppression with intravenous methylprednisolone would further impair response to infection and plasma exchange was considered hazardous due to CD4 T lymphocytes depletion, re-increase of prednisolone to 40 ​mg per day and immunoglobulin infusion therapy (0.4 ​mg/kg per day for 5 days) constituted the initial choice. Thereon, the patient slowly improved, and, on day 27, EEG displayed well-structured alpha activity, without paroxysmal activity nor ‘delta brush’ pattern (Fig. 2B, Supplementary Data). Later on, prednisolone was progressively stepped-down, with no clinical worsening. Six weeks of induction therapy were completed and maintenance treatment with fluconazole was initiated given confirmed cultural negativity. The patient was discharged on day 51 with 10 ​mg of prednisolone per day, with total clinical resolution and imaging improvement.

Four months after discharge, the patient remained neurologically asymptomatic but anti-NMDAR antibodies were still present on CSF (negative in serum), with a high IgG level (1.03 ​μmol/L) and seven CSF-restricted oligoclonal bands. The patient was re-started on intravenous immunoglobulin (1 ​g/kg per month). Infliximab was initiated at seven months after discharge, due to sarcoid progression and very low CD4 counts. At 12-months follow-up, the patient displays no neurologic symptoms, with CD4 elevation until 165/mm^3^, maintaining fluconazole secondary prophylaxis.

## 3 Discussion and conclusions

We report a case of concomitant cryptococcal meningitis and anti-NMDAR encephalitis in a sarcoidosis adult patient. Recent experimental studies have shown a significant inflammatory response in cryptococcal CNS disease, particularly in non-HIV cases, driving tissue damage (Neal et al., 2017; Panackal et al., 2015). Hereby, we consider the hypothesis of an infectious immune-mediated mechanism triggering anti-NMDAR antibodies production.

Considering the patient’s background, there were several initial diagnostic possibilities: in a patient with sarcoidosis, neurological involvement (neurosarcoidosis) must be considered; in a patient under immunosuppression, risk of infection must be carefully addressed. A preliminary diagnosis of neurosarcoidosis determined an increase in steroid dosing, which led to clinical deterioration. Differentiation between cryptococcal meningitis and neurosarcoidosis can be challenging, as they both often present with symptoms of chronic meningitis and can be complicated by hydrocephalus (Leonhard et al., 2016). CSF abnormalities can be similar, with a mild pleocytosis and elevated protein, as well as hypoglycorrachia to some degree (Leonhard et al., 2016). In neurosarcoidosis, any portion of the central or peripheral nervous system can be affected, and the IgG index can be elevated, with presence of oligoclonal bands in CSF, as we saw in our patient. However, for its diagnosis, an intercurrent infection must be excluded. Furthermore, the absence of response to the rise in corticotherapy dosing (on the contrary, the patient deteriorated) makes neurosarcoidosis unlikely.

Cryptococcal meningitis is a rare complication of sarcoidosis associated with CD4 lymphopenia, which diagnosis is often delayed or missed (Leonhard et al., 2016). In our patient, cell-mediated immunity was already impaired before steroid treatment. Cryptococcosis alone could not explain this clinical picture; the illness exhibited a biphasic course, which is not typical in cryptococcal meningitis. We postulate that our patient could initially be suffering from chronic meningitis due to cryptococcal infection, as he first presented with characteristic symptoms and aggravated with intensified immunosuppression, and that this infection triggered the production of anti-NMDAR antibodies, leading to the subsequent development of neuropsychiatric symptoms characteristic of this condition. He was medicated with psychotropic drugs, again with no improvement, and it is not unusual to anti-NMDAR encephalitis to be confounded with psychiatric disorders (Dalmau et al., 2008, 2011; Hermans et al., 2017). A clinical response to antifungal treatment can be inferred by CSF cytochemical improvement and normalization of CSF pressure. However, we observed a progressive depression of consciousness under effective therapy. EEG monitoring showed generalized slowing with occasional delta-brush pattern, which is consistent with anti-NMDAR encephalitis (Steriade et al., 2018). The particular ‘extreme delta brush’ pattern, initially described as pathognomonic of this disorder, was not present, but it is usually seen in only 16–33% of patients (Venkatesan and Adatia, 2017). Nonspecific slowing of brain activity is typically seen and focal electrographic seizures can also be present (Dalmau et al., 2011; Fischer et al., 2016; Guasp and Dalmau, 2018; Venkatesan and Adatia, 2017; Zhang et al., 2017). Above all, we only observed neurological improvement after immune therapy and a new rise in corticoid dosing. A diagnosis of anti-NMDAR encephalitis was established based on the diagnostic criteria (Graus et al., 2016): rapid onset (less than 3 months) of abnormal (psychiatric) behavior, cognitive and speech dysfunction, decreased level of consciousness and autonomic dysfunction; EEG showing ‘delta brush’ pattern and focal paroxysmal activity, with slow disorganized activity; CSF with pleocytosis and oligoclonal bands, with the presence of IgG anti-NMDA antibodies specifically directed to the NR1 subunit.

CD4 lymphopenic sarcoidosis phenotype is usually resistant to conventional therapies and responsive to anti-TNF therapy (Crouser et al., 2010). In these patients, infliximab may normalize peripheral CD4 T-cell depletion and improve clinical disease manifestations (Crouser et al., 2010). CD4 counts should be frequently evaluated and prophylaxis prescribed accordingly – primary prophylaxis with cotrimoxazole for *Pneumocystis* pneumonia and secondary prophylaxis with fluconazole in case of previous cryptococcal meningitis.

Lastly, it is of notice that our report has several limitations. First, it does not establish proven causation between infection by *Cryptococcus* and CNS autoimmunity. Mechanistic studies linking infections with anti-NMDAR encephalitis are lacking (Venkatesan and Benavides, 2015), and the presence of co-incident infection alone with an autoimmune process is not sufficient to establish causality (Venkatesan and Benavides, 2015). Henceforth, studies are needed to firmly establish such a link. Secondly, this study is limited by the absence of a CSF sample from the pre-illness phase or even from the first phase of the illness, in which we speculate there would be no anti-NMDAR antibodies production. Nevertheless, since this autoimmune encephalitis can be fatal and its prognosis can be significantly improved by prompt treatment, the knowledge of new probable triggers is of particular importance to enhance its recognition. Investigation of other related or causal triggers may allow a new understanding of anti-NMDAR encephalitis and facilitate its early diagnosis.

## Ethical standards

The patient has agreed to this publication by written consent.

## Declaration of competing interest

None.

## References

[bib1] Adams T.N., Gibson M. (2016). Cryptococcal meningitis in a patient with sarcoidosis. Proceedings.

[bib2] Arboleya S., Clemente A., Deng S., Bedmar M., Salvador I., Herbera P., Cunill V., Vives-Bauza C., Haro J.M., Canellas F., Julia M.R. (2016). Anti-NMDAR antibodies in new-onset psychosis. Positive results in an HIV-infected patient. Brain Behav. Immun..

[bib3] Armangue T., Leypoldt F., Malaga I., Raspall-Chaure M., Marti I., Nichter C., Pugh J., Vicente-Rasoamalala M., Lafuente-Hidalgo M., Macaya A., Ke M., Titulaer M.J., Hoftberger R., Sheriff H., Glaser C., Dalmau J. (2014). Herpes simplex virus encephalitis is a trigger of brain autoimmunity. Ann. Neurol..

[bib4] Armangue T., Titulaer M.J., Málaga I., Bataller L., Gabilondo I., Graus F., Dalmau J. (2013). Pediatric Anti-NMDAR encephalitis-Clinical analysis and novel findings in a series of 20 patients. J. Pediatr..

[bib5] Baughman R.P., Lower E.E. (2005). Fungal infections as a complication of therapy for sarcoidosis. QJM : Mon. J. Assoc. Phys..

[bib6] Bernard C., Maucort-Boulch D., Varron L., Charlier C., Sitbon K., Freymond N., Bouhour D., Hot A., Masquelet A.C., Valeyre D., Costedoat-Chalumeau N., Etienne M., Gueit I., Jouneau S., Delaval P., Mouthon L., Pouget J., Serratrice J., Brion J.P., Vaylet F., Bremont C., Chennebault J.M., Jaffuel S., Broussolle C., Lortholary O., Seve P. (2013). Cryptococcosis in sarcoidosis: cryptOsarc, a comparative study of 18 cases. QJM : Mon. J. Assoc. Phys..

[bib7] Crouser E.D., Lozanski G., Fox C.C., Hauswirth D.W., Raveendran R., Julian M.W. (2010). The CD4+ lymphopenic sarcoidosis phenotype is highly responsive to anti-tumor necrosis factor-{alpha} therapy. Chest.

[bib8] Dalmau J., Gleichman A.J., Hughes E.G., Rossi J.E., Peng X., Lai M., Dessain S.K., Rosenfeld M.R., Balice-Gordon R., Lynch D.R. (2008). Anti-NMDA-receptor encephalitis: case series and analysis of the effects of antibodies. Lancet Neurol..

[bib9] Dalmau J., Lancaster E., Martinez-Hernandez E., Rosenfeld M.R., Balice-Gordon R. (2011). Clinical experience and laboratory investigations in patients with anti-NMDAR encephalitis. Lancet Neurol..

[bib10] Dhote R., Abad S., Valeyre D. (2009). [The infectious complications of sarcoidosis]. Presse Med..

[bib11] Fischer C.E., Golas A.C., Schweizer T.A., Munoz D.G., Ismail Z., Qian W., Tang-Wai D.F., Rotstein D.L., Day G.S. (2016). Anti N-methyl-D-aspartate receptor encephalitis: a game-changer?. Expert Rev. Neurother..

[bib12] Gable M.S., Gavali S., Radner A., Tilley D.H., Lee B., Dyner L., Collins A., Dengel A., Dalmau J., Glaser C.A. (2009). Anti-NMDA receptor encephalitis: report of ten cases and comparison with viral encephalitis. Eur. J. Clin. Microbiol. Infect. Dis..

[bib13] Gable M.S., Sheriff H., Dalmau J., Tilley D.H., Glaser C.A. (2012). The frequency of autoimmune N-methyl-D-aspartate receptor encephalitis surpasses that of individual viral etiologies in young individuals enrolled in the California Encephalitis Project. Clin. Infect. Dis..

[bib14] Girard N., Cottin V., Hot A., Etienne-Mastroianni B., Chidiac C., Cordier J.F. (2004). [Opportunistic infections and sarcoidosis]. Rev. Mal. Respir..

[bib15] Graus F., Titulaer M.J., Balu R., Benseler S., Bien C.G., Cellucci T., Cortese I., Dale R.C., Gelfand J.M., Geschwind M., Glaser C.A., Honnorat J., Hoftberger R., Iizuka T., Irani S.R., Lancaster E., Leypoldt F., Pruss H., Rae-Grant A., Reindl M., Rosenfeld M.R., Rostasy K., Saiz A., Venkatesan A., Vincent A., Wandinger K.P., Waters P., Dalmau J. (2016). A clinical approach to diagnosis of autoimmune encephalitis. Lancet Neurol..

[bib16] Guasp M., Dalmau J. (2018). Encephalitis associated with antibodies against the NMDA receptor. Med. Clín. (Barc).

[bib17] Hermans T., Santens P., Matton C., Oostra K., Heylens G., Herremans S., Lemmens G.M.D. (2017). Anti-NMDA receptor encephalitis: still unknown and underdiagnosed by physicians and especially by psychiatrists?. Acta Clin. Belg..

[bib18] Jamilloux Y., Valeyre D., Lortholary O., Bernard C., Kerever S., Lelievre L., Neel A., Broussolle C., Seve P. (2015). The spectrum of opportunistic diseases complicating sarcoidosis. Autoimmun. Rev..

[bib19] Leonhard S.E., Fritz D., van de Beek D., Brouwer M.C. (2016). Cryptococcal meningitis complicating sarcoidosis. Medicine.

[bib20] Liu C.Y., Zhu J., Zheng X.Y., Ma C., Wang X. (2017). Anti-N-Methyl-D-aspartate receptor encephalitis: a severe, potentially reversible autoimmune encephalitis. Mediat. Inflamm..

[bib21] Lynch D.R., Rattelle A., Dong Y.N., Roslin K., Gleichman A.J., Panzer J.A. (2018). Anti-NMDA receptor encephalitis: clinical features and basic mechanisms. Adv. Pharmacol..

[bib22] Ma J., Zhang T., Jiang L. (2017). Japanese encephalitis can trigger anti-N-methyl-D-aspartate receptor encephalitis. J. Neurol..

[bib23] Mehrany K., Kist J.M., Gibson L.E. (2002). Cryptococcal infection in sarcoidosis. Int. J. Dermatol..

[bib24] Miyara M. (2006). The immune paradox of sarcoidosis and regulatory. T cells.

[bib25] Morell F., Levy G., Orriols R., Ferrer J., De Gracia J., Sampol G. (2002). Delayed cutaneous hypersensitivity tests and lymphopenia as activity markers in sarcoidosis. Chest.

[bib26] Neal L.M., Xing E., Xu J., Kolbe J.L., Osterholzer J.J., Segal B.M., Williamson P.R., Olszewski M.A. (2017). CD4(+) T cells orchestrate lethal immune pathology despite fungal clearance during Cryptococcus neoformans meningoencephalitis. mBio.

[bib27] Panackal A.A., Wuest S.C., Lin Y.C., Wu T., Zhang N., Kosa P., Komori M., Blake A., Browne S.K., Rosen L.B., Hagen F., Meis J., Levitz S.M., Quezado M., Hammoud D., Bennett J.E., Bielekova B., Williamson P.R. (2015). Paradoxical immune responses in non-HIV cryptococcal meningitis. PLoS Pathog..

[bib28] Patarata E., Bernardino V., Martins A., Pereira R., Loureiro C., Moraes-Fontes M.F. (2016). Anti-N-Methyl-D-Aspartate receptor encephalitis in HIV infection. Case Rep. Neurol..

[bib29] Peret G., Picard A., Corneloup O., Begueret H., Raherison-Semjen C. (2014). [Cryptococcal infection and sarcoidosis: a coincidence?]. Rev. Pneumol. Clin..

[bib30] Prüss H., Finke C., Höltje M., Hofmann J., Klingbeil C., Probst C., Borowski K., Ahnert-Hilger G., Harms L., Schwab J.M., Ploner C.J., Komorowski L., Stoecker W., Dalmau J., Wandinger K.P. (2012). N-Methyl-d-Aspartate receptor antibodies in herpes simplex encephalitis. Ann. Neurol..

[bib31] Riha R.L., Allen R.K. (2004). Cryptococcosis and sarcoidosis: strange bed-fellows. A report of five cases. Sarcoidosis, vasculitis, and diffuse lung diseases. Off. J. WASOG.

[bib32] Ross J.J., Katz J.D. (2002). Cryptococcal meningitis and sarcoidosis. Scand. J. Infect. Dis..

[bib33] Schabitz W.R., Rogalewski A., Hagemeister C., Bien C.G. (2014). VZV brainstem encephalitis triggers NMDA receptor immunoreaction. Neurology.

[bib34] Steriade C., Hantus S., Moosa A.N.V., Rae-Grant A.D. (2018). Extreme delta - with or without brushes: a potential surrogate marker of disease activity in anti-NMDA-receptor encephalitis. Clin. Neurophysiol..

[bib35] Venkatesan A., Adatia K. (2017). Anti-NMDA-receptor encephalitis: from bench to clinic. ACS Chem. Neurosci..

[bib36] Venkatesan A., Benavides D.R. (2015). Autoimmune encephalitis and its relation to infection. Curr. Neurol. Neurosci. Rep..

[bib37] Yanardag H., Pamuk G.E., Karayel T., Demirci S. (2002). Bone marrow involvement in sarcoidosis: an analysis of 50 bone marrow samples. Haematologia.

[bib38] Zhang Y., Liu G., Jiang M.D., Li L.P., Su Y.Y. (2017). Analysis of electroencephalogram characteristics of anti-NMDA receptor encephalitis patients in China. Clin. Neurophysiol..

